# A Novel Lightweight Algorithm for Sonar Image Recognition

**DOI:** 10.3390/s25113329

**Published:** 2025-05-26

**Authors:** Gang Wan, Qi He, Qianqian Zhang, Hanren Wang, Huanru Sun, Xinnan Fan, Pengfei Shi

**Affiliations:** 1 College of Water Conservancy and Hydropower Engineering, Hohai University, Nanjing 210024, China; wan_gang@ctg.com.cn; 2 China Yangtze Power Co., Ltd., Yichang 443002, China; 3 College of Artificial Intelligence and Automation, Hohai University, Changzhou 213200, China; 221320020003@hhu.edu.cn (Q.H.); 241614010101@hhu.edu.cn (Q.Z.); 231322020005@hhu.edu.cn (H.W.); 4 College of Information Science and Engineering, Hohai University, Changzhou 213200, China; sunhr@hhu.edu.cn (H.S.); fanxn@hhu.edu.cn (X.F.)

**Keywords:** sonar images, object recognition, MobileViT, convolutional neural networks, feature extraction

## Abstract

Sonar images possess characteristics such as low resolution, high noise, and blurred edges. Utilizing CNNs would lead to problems such as inadequate target recognition accuracy. Moreover, due to their larger sizes and higher computational requirements, existing CNNs face deployment issues in embedded devices. Therefore, we propose a sonar image recognition algorithm optimized for the lightweight algorithm, MobileViT, by analyzing the features of sonar images. Firstly, the MobileViT block is modified by adding and redesigning the jump connection layer to capture more important features of sonar images. Secondly, the original 1 × 1 convolution is replaced with the redesigned multi-scale convolution Res2Net in the MV2 module to enhance the ability of the algorithm to learn global and local features. Finally, the IB loss is applied to address the imbalance of sample categories in the sonar dataset, assigning different weights to the samples to improve the performance of the network. The experimental results show that several proposed improvements have improved the accuracy of sonar image recognition to varying degrees. At the same time, the proposed algorithm is lightweight and can be deploy on embedded devices.

## 1. Introduction

Because the convolution neural network (CNN) [1,2] has obtained good results in the field of optical image recognition, researchers have gradually introduced it into sonar images [3,4]. However, due to the complexity of the marine environment, the acquired sonar images are characterized by low resolution, high noise, and fuzzy edges [5,6,7], and the direct application of the existing CNN network to sonar images will lead to the problems of insufficient feature extraction and low recognition accuracy.

In 2012, the proposal of AlexNet [8] indicated a new stage in the development of convolution neural networks. After years of development, CNN has become very mature both in the design of the algorithm structure and the tuning of hardware and software. However, due to the limitation of convolution, CNN networks cannot capture image global information well. With the Vision Transformer (ViT) proposed in 2020 [9], researchers have found that the Transformer architecture shows great potential in the field of computer vision. Inspired by this, more and more researchers have been devoted to studying the application of the Transformer to the field of image processing in recent years, and many new algorithms have been proposed. Although the Transformer refreshes the task metrics in the vision domain, the large parameters and high arithmetic requirements of the Transformer [10] make it difficult to deploy on mobile. To address this problem, some researchers have started to explore lightweight Transformer architectures. For example, Swin_Transformer [11], proposed in 2021, and its improved version Swin_Transformerv2 [12] perform better and are lighter than ViT on public datasets. However, compared with lightweight CNN-based algorithms, Transformer-based algorithms still have a significant gap in both parameters and inference speed. To fully combine the advantages of CNN and Transformer architectures and make the algorithm more lightweight and efficient, more and more scholars have invested in the research of hybrid CNN and Transformer architectures.

In 2022, Apple proposed MobileViT, a lightweight algorithm that can be used for mobile devices, by combining the advantages of CNN and ViT [13]. The core idea of the MobileViT is to use the Transformer as a convolution to learn a global representation of the image. MobileViT enables algorithms with more powerful information extraction by replacing the local information processing of CNN with the global information processing of Transformer [14]. Thus, it is the first lightweight ViT that can achieve similar results as lightweight CNNs on the ImageNet-1k dataset. CNNs are computationally efficient and have relatively simple algorithms. It is also characterized by spatial translation invariance and low sensitivity to data enhancement techniques. However, it can only capture localized information due to the limitations of the sensory field. The transformer can establish global dependencies with the advantage of the adaptive weighted sum of input feature map information. However, it is computationally complex and computationally expensive. The MobileViT network is a lightweight end-side network architecture that combines the advantages of CNN and Transformer. In this network, CNN is responsible for extracting local features and Transformer is responsible for extracting global features. By using a hybrid of CNN and Transformer, local and global information can be modeled in the input tensor with fewer parameters. Therefore, the MobileViT algorithm is selected as the baseline for the sonar target recognition algorithm in this paper. However, this algorithm is designed for optical images where foreground and background features are easily distinguishable. Applying the MobileViT algorithm directly to sonar images where the target and background are difficult to distinguish would result in insufficient feature extraction capabilities of the network. Sonar images are noisy and have low resolution, and the target is not clearly distinguished from the background. A combination of both deep detail features and global features is needed to capture the rich multidimensional information of sonar images and improve recognition accuracy. Therefore, the MobileViT algorithm needs to be improved for the characteristics and distributional differences of sonar image samples.

However, traditional image recognition algorithms face challenges due to the complex underwater environment, low-resolution sonar image data, and small sample sizes, which result in difficulties in achieving accurate recognition. Therefore, there is a need to develop more effective methods to improve the performance of sonar image target recognition [15]. Existing target recognition algorithms for sonar images are focused on improving their detection accuracy. Ruan et al. [16] proposed a simple and effective neural network attention module to improve the classification accuracy and classification efficiency of sonar images by extracting information from input features with different scales. Cheng et al. [17] proposed a repeated attention mechanism that effectively combines multi-scale features to increase recognition accuracy when using multi-domain datasets. Shang and Liu created a target detection method combining wavelet packet decomposition with a sophisticated CNN to detect small targets in noisy, weak-echo underwater conditions [18]. Song et al. [19] proposed a self-cascading neural network based on an improved convolutional neural network that can take into account both global and local feature information, which improves the performance of the network. However, many underwater tasks, such as underwater vehicle navigation and underwater environmental monitoring, rely on sonar systems mounted on resource-constrained mobile devices. Therefore, there is an urgent need for an algorithm capable of processing complex underwater images and running efficiently under resource-constrained conditions. Although significant progress has been made in sonar image recognition using deep learning algorithms, these models typically demand substantial computational resources, making it challenging to achieve practical deployment. Therefore, for the characteristics of sonar images, we improve and propose an underwater sonar image target recognition algorithm based on MobileViT. In our study, we introduce skip connections in the MobileViT block and incorporate the improved Res2Net structure into the MV2 (MobileNet V2) module. These enhancements enable our algorithm to more effectively capture the crucial features extracted from sonar images, which are crucial for improving target recognition accuracy. Additionally, we introduce the IB loss to replace the cross-entropy loss function. This adjustment successfully addresses the common issue of class imbalance in underwater sonar data, significantly enhancing the model’s ability to recognize minority class targets. The algorithm can accurately recognize common scenes in the ocean such as persons, fish, and shipwrecks, while having a small computational overhead for underwater detection. Experiments show that this algorithm has higher recognition accuracy than other algorithms, is suitable for practical applications, and can be deployed on embedded devices.

The remainder of this paper is organized as follows. Section 2 reviews the existing research and related work in the domain of sonar image recognition. Section 3 shows the proposed improvement strategy. Section 4 describes the experimental environment and sonar image dataset employed. The outcomes of the experiments are presented in Section 5, followed by a discussion of the findings and an examination of the study’s limitations in Section 6. Finally, Section 7 offers a conclusion of the work and provides future work.

## 2. Related Work

In the field of optical image processing, convolutional neural networks (CNNs) based on deep learning have shown significant development momentum. This greatly promotes the advancement of image recognition and processing technology [20,21]. Leveraging large-scale datasets, CNNs can learn image features to automatically predict target characteristics, effectively achieving precise target recognition. Furthermore, for vision-related tasks, model parameters learned from specific datasets can be applied to new datasets using transfer learning techniques [22]. Therefore, CNN-based algorithms suitable for optical images can be adapted to the requirements of sonar image processing through appropriate optimization and adjustment. However, due to the significant differences in sample characteristics and distribution between sonar and optical images, sonar image processing requires specific optimizations and adjustments to the network structure. Particularly in the field of sonar image processing, where the number of training samples is limited, directly using existing CNN models may present challenges and may not achieve ideal processing results. In response to this challenge, scholars have proposed the use of transfer learning [23] techniques for computer vision tasks, which involves adding additional layers to existing models and fine-tuning with smaller datasets, thus achieving higher recognition accuracy in the field of sonar images. For instance, Wang et al. [24] proposed a method for enhancing the classification accuracy of underwater sonar images by performing style transfer between optical images and side-scan sonar (SSS) images. A transfer learning approach was utilized, in which a backbone network was trained on a large-scale optical dataset and then fine-tuned on a smaller SSS image dataset for the head network. Xu et al. [25] developed a multi-feature fusion self-attention network (MFSANet) for the generation of novel categories of SSS images, transforming the zero-shot problem into a regular supervised learning task. This method effectively boosted the recognition accuracy of SSS images by incorporating a self-attention mechanism and contrastive loss. Long et al. [26] designed an object detection network for forward-looking sonar (FLS) images, named UFIDNet, which considered reducing speckle noise caused by scattering and leveraging scene priors to enhance detection performance. Experimental results demonstrated that UFIDNet outperformed several state-of-the-art detectors on two real-world FLS datasets, exhibiting higher recognition accuracy and faster processing speed.

## 3. Proposed Work

### 3.1. MobileViT

MobileViT comes in three different configurations: MobileViT-S (small), MobileViT-XS (extra small), and MobileViT-XXS (extra-extra small). We tested the computational complexity and parameter count of models of different sizes on the sonar dataset to measure the complexity of the algorithm. The overall framework of MobileViT can be divided into five layers. The difference between models of different sizes lies in the number of channels in the output feature maps of these five layers. The results of the tests are presented in Table 1. Among these configurations, MobileViT-XXS has the smallest parameter count and demands the least computational power, while the MobileViT-S model has the largest parameter count and requires the highest computational power. In comparison, the MobileViT-XS size and its computational requirements are intermediate. Considering the size of the models and the constraints of hardware devices in practical scenarios, we chose MobileViT-XS as the baseline.

**Figure 1:**
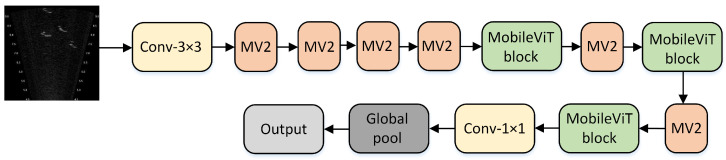
The network architecture of MobileViT.

The structure of MobileViT is shown in Figure 1. In Figure 1, one can observe that MobileViT primarily comprises regular convolutions, a MobileViT block, MV2, global pooling, and fully connected layers.

**Figure 2:**
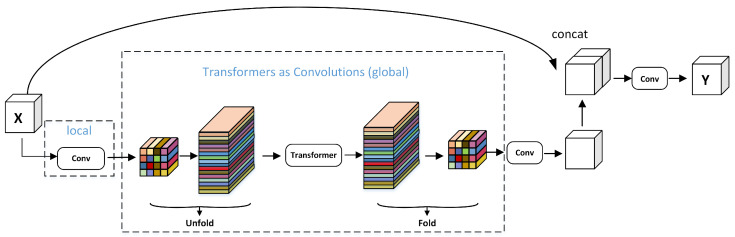
The structure of the MobileViT block.

The MobileViT block is capable of learning global representations from various perspectives, effectively enhancing the ability of the algorithm to encode both local and global information. Its standard convolution involves three operational steps: unfold, local processing, and folding. The MobileViT block uses Transformer to conduct global modeling to replace local modeling in convolution, thus making the MobileViT block have the properties of both CNN and ViT, enabling it to better learn feature representation using fewer parameters and simpler training. The structure of the MobileViT block is shown in Figure 2. First, the feature map is put through the convolutional feature extraction module for local feature modeling. Second, the global feature modeling is performed through the structure composed of the unfolding module, the Transformer module, and folding module (UTF). Third, the shortcut branch splices the input feature maps in the channel dimension. Finally, the output features are obtained by feature fusion through a convolutional layer with a convolutional kernel size of 3 × 3.

**Figure 3:**
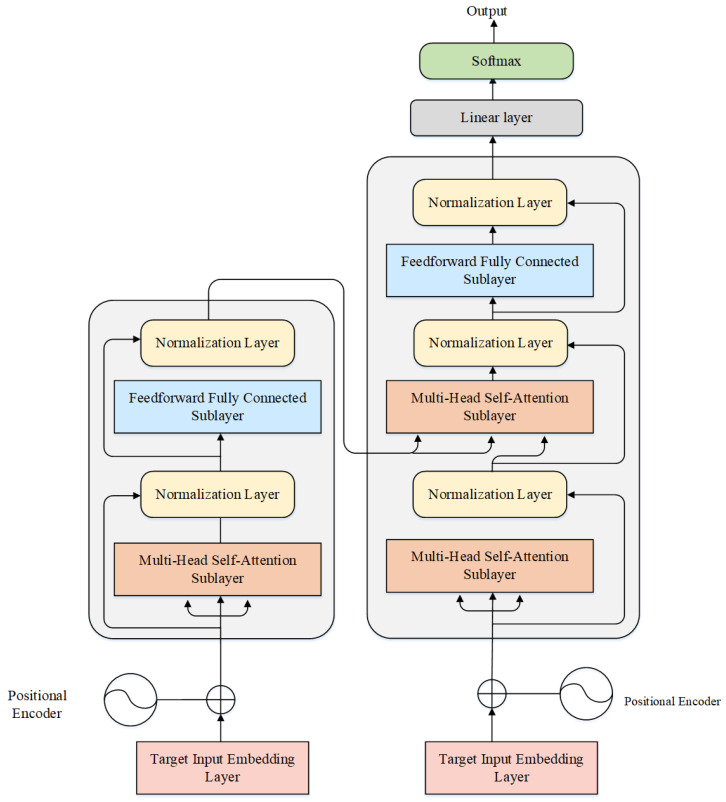
The architecture of the Transformer.

The Transformer structure is shown in Figure 3. The Transformer architecture consists of an encoder and a decoder, each comprising multiple layers. Each layer is composed of a multi-head self-attention mechanism and a feedforward neural network. The gray part on the left half in the figure is the encoder part, and the gray part on the right half is the decoder part. The multi-head self-attention layer consists of multiple self-attention sub-modules. It enables each position in the input sequence to interact with others, thereby comprehensively capturing global information within the input sequence. The feedforward neural network sublayer processes features at each position through fully connected layers and activation functions, thereby enhancing the model’s expressive capacity. The normalization layer comprises residual connections and normalization operations. Residual connections aid in preventing network performance degradation, while normalization operations are used to normalize the activation values of each layer.

### 3.2. Skip Connection

The MobileViT block, as an important module in the MobileViT, plays an important role in the feature extraction and fusion of images. The algorithm needs to continuously pass the convolution operation to enrich the semantic information of the features for the subsequent recognition task, considering the resolution of sonar images is low. Therefore, multiple layers of feature extraction may result in the network losing shallow-level details. This paper enriches the detailed features of sonar targets in the algorithm by adding and modifying skip connections. Enriched detailed features will help improve the accuracy of the algorithm.

**Figure 4:**
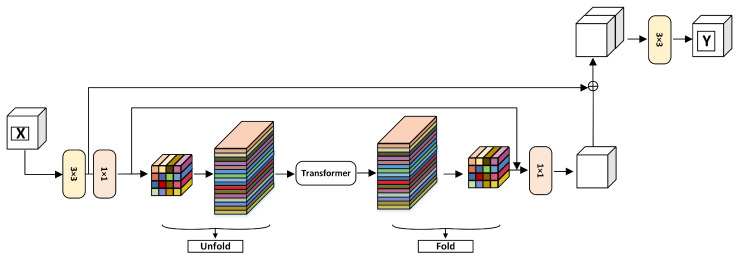
Improved MobileViT block.

The improved MobileViT block framework is shown in Figure 4. For the input $X\in R^{H\times W\times C}$, initial feature extraction is first performed on the input using 3 × 3 and 1 × 1 convolution to obtain the output features $X_{L}\in R^{H\times W\times C}$. The 3 × 3 convolution can capture the local spatial information of the image and the 1 × 1 convolution is responsible for projecting the features into the high-dimensional space. Then, to obtain the global representation, the algorithm first unfolds the features $X_{L}$ into the N non-overlapping patch to obtain the feature $X_{U}\in R^{P\times N\times d}$, where P=w×h and N=H×W/P and where *h*, *w* are the height and width of the patch, respectively. Then, the feature $X_{G}\in R^{P\times N\times d}$ is obtained after modeling by the Transformer. This preserves both the order of the patch and the spatial order of the pixels within each patch. Next, the feature $X_{G}\in R^{P\times N\times d}$ is folded by the fold operation to obtain the feature $X_{F}\in R^{H\times W\times d}$. Because the algorithm encodes the local information of the n×n region for the feature $X_{U}\left(p\right)$ and the global information of the p-th position of $X_{G}\left(p\right)$, $X_{G}$ can perceive the global information in *X*. Therefore, the size of the overall receptive field of the algorithm is H × W.

**Figure 5:**
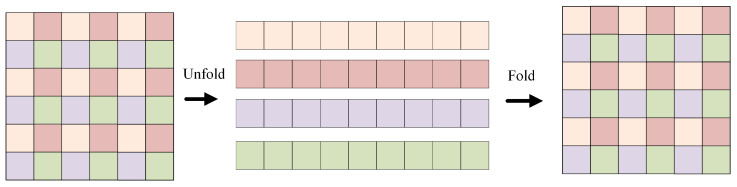
Unfold and fold process.

The UTF structure belongs to the global perception module, and its effect is equivalent to the self-attention calculation module. Its specific calculation process is shown in Figure 5. Firstly, the feature map is divided into many patches. Each patch has a size of 2 × 2, consisting of 4 pixels. During attention calculation, each token is only computed with blocks of the same color. This effectively reduces computational complexity. In the algorithm, the input feature *X* has a size of W × H × C. After undergoing feature extraction with a 3 × 3 convolution and a 1 × 1 convolution, its channel number becomes d. After the UTF structure, its size remains unchanged. Subsequently, a 1 × 1 convolution is applied, reducing the channel number of the feature to *C*. The final output feature *Y* of the module has the same size as the input *X*.

In this paper, we improve the MobileViT block by adding two skip connection layers. First, a 3 × 3 convolution is performed on the input feature *X*. The result of the convolution is then concatenated with the features after the UTF structure. This is the first skip connection, where the output channels are doubled compared with the input. A 3 × 3 convolution is then used to reduce the number of channels while further extracting deep semantic features. The second skip connection is a concatenation of the result after the first 1 × 1 convolution with the result after the fold operation. Subsequently, 1 × 1 convolution is introduced for channel dimensional reduction so that the channel dimension is equal to the input channel *C*. By adding skip connections, the depth information of the network is enriched. Moreover, skip connections also enhance the ability of the network to learn useful sonar features.

### 3.3. Multi-Scale Feature Fusion Module

**Figure 6:**
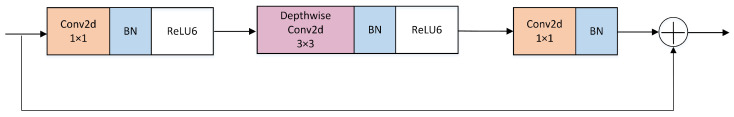
The architecture of MV2.

The structure of the MV2 module in MobileViT is the same as the structure of the inverted residual block in MobiletNetV2, which is shown in Figure 6. Initially, the feature maps undergo expansion through a 1 × 1 convolutional layer to increase their depth. The purpose of this step is to provide the network with sufficient expressive capacity to handle complex feature structures. Subsequently, the expanded feature maps are processed through depthwise convolutional layers. Depthwise convolution significantly reduces the number of parameters and computational load of the network. Following this, the depth of the expanded feature maps is reduced back to the original depth through another 1 × 1 convolutional layer. No activation function is applied at this stage to minimize information loss. Additionally, to further enhance network performance, the MV2 module incorporates residual connections. This design facilitates information flow and effectively improves the training stability and performance of the network.

In many visual tasks, multi-scale feature extraction plays a very important role in improving the information-capturing ability of images. As convolutional neural networks continue to advance, researchers are moving closer to improving the ability of the algorithms to characterize features at multiple scales to achieve performance improvements. To obtain feature extraction networks with better characterization capabilities, Res2Net has been proposed in the literature [27]. Inspired by grouped convolutions, Res2Net replaces the 3 × 3 convolution in the bottleneck block with several small convolutional groups. These convolutional groups are connected using a hierarchical residue-like style to increase the scale representation of the output features. The hierarchical residue-like connection is defined as shown in the following equation:

(1) $$y_{i}=\left\{\begin{array}{cc} x_{i}, & i=1\\ K_{i}\left(x_{i}\right), & i=2\\ K_{i}\left(x_{i}+y_{i-1}\right), & 2<i\leq s \end{array}\right.,$$

where $x_{i}$ denotes the input of the *i* convolutional group, $y_{i}$ denotes the output of the *i* convolutional group, $K_{i}$ denotes the *i* convolutional operation performed by the *i* convolutional group, and *s* denotes the number of convolutional groups.

**Figure 7:**
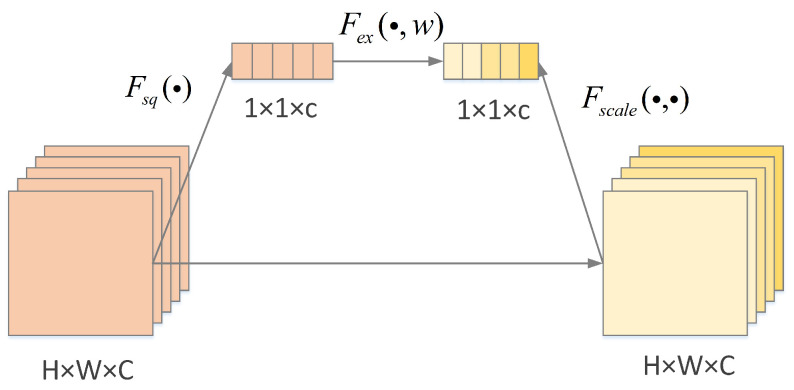
The architecture of the SE block.

In this paper, we improve Res2Net by removing residual connections and adding a squeeze-and-excitation (SE) block [28] after convolution. The SE block can adaptively recalibrate the characteristic responses of channels through inter-correlations between channels. Such an improvement enables the Res2Net structure to further enhance the useful features and suppress the useless features of the sonar image. The structure of one of the SE block modules is shown in Figure 7. The SE block begins by performing global average pooling to acquire the global information of each channel within the feature map. Subsequently, a fully connected neural network is employed to learn and adjust the weight coefficients for each channel. Following this, the original feature map channels are recalibrated based on these weight coefficients. Finally, the original input is combined with the feature map adjusted by the learned weights through residual connections. This preserves the original feature information and accelerates training speed. The implementation of this structure effectively enhances the convolutional neural network’s ability to capture crucial information, thereby significantly improving model performance. Its calculation for each feature channel is as follows:

(2) $$g_{c}=F_{sq}\left(f_{c}\right)=\frac{1}{H\times W}\sum_{i=1}^{H}\sum_{j=1}^{W}f_{c}\left(i,j\right),$$

where $g_{c}$ is the output of the channel, $f_{c}$ is the input of the *c* channel, *H* is the height of the feature map, *W* is the width of the feature map, and *T* is the Squeeze operation function. This is followed by the excitation operation to capture the correlation between the channels and generate the weights for each channel by training, which is computed as follows:

(3) $$U_{c}=F_{scale}\left(f_{c},e_{c}\right)=e_{c}\cdot f_{c},$$

where $F_{scale}\left(\cdot \right)$ is the fusion function of weights and input features, and the final output feature is $U=[U_{1},U_{2},\cdots ,U_{c}]$.

**Figure 8:**
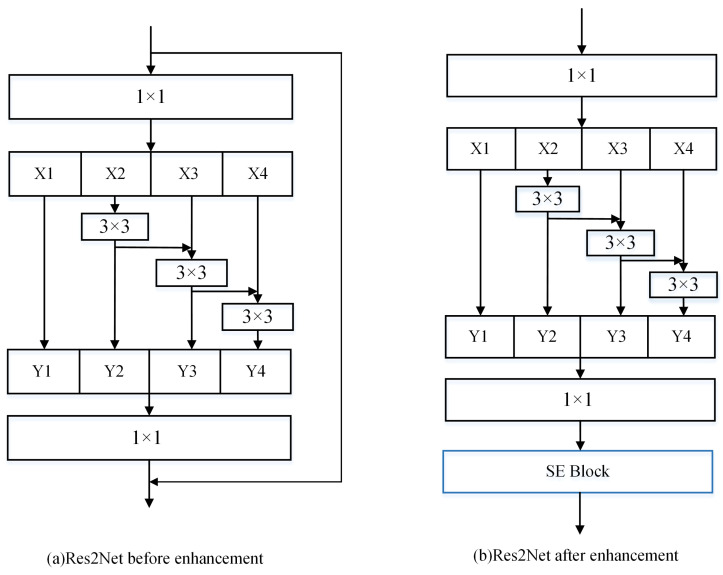
Res2Net and its improvements.

**Figure 9:**
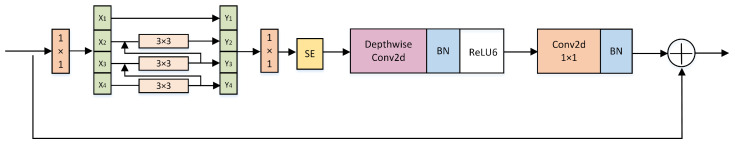
Improved architecture of MV2.

The Res2Net structure, as well as the improved structure, is shown in Figure 8. Figure 8a illustrates the original structure of the Res2Net block. In this architecture, features are processed through multiple pathways simultaneously, with each pathway conducting feature extraction at different scales. Subsequently, these features are reassembled through a feature fusion module to obtain a richer and more diverse representation. Figure 8b depicts the enhanced version of the Res2Net block. In this improved iteration, residual connections are omitted, and an SE block is introduced after the feature fusion module. The enhanced Res2Net block not only enhances the feature representation capability but also exhibits higher efficiency and better generalization. To obtain multi-scale features of the sonar image as well as to obtain deeper semantic information about the sonar image, we then improved the MV2 module, which is reused in the algorithm. The improved MV2 module structure is shown in Figure 9. In the original MobileViT algorithm, the MV2 module uses the 1 × 1 convolutional layer for feature extraction. However, the simple 1 × 1 convolutions primarily adjust the number of channels and cannot capture larger-scale features in sonar images. It has limited receptive fields and weak feature extraction capability. Therefore, we optimize the first 1 × 1 convolution in the MV2 module. Compared with the original MV2 module illustrated in Figure 6, we replace the 1 × 1 convolutions with the enhanced Res2Net block. This improvement allows the algorithm to not only represent multi-scale features at a finer granularity level but also increase the receptive field of each network layer.

### 3.4. Loss Function

Recognition algorithms commonly use the cross-entropy loss function to measure the recognition results. As an important piece of equipment for detecting the sea, sonar is expensive to use, and the images acquired may involve military secrets, such as torpedoes and other military targets, resulting in an insufficient number of samples of sonar images and an uneven distribution. Therefore, the number of samples in the dataset is uneven. Moreover, the convolutional neural network training process will be more biased towards categories with a large number of samples, so categories with a large number of samples tend to perform better than those with a small number of samples. The original MobileViT uses a cross-entropy loss function, which cannot solve this problem better. For this reason, to address the sample imbalance problem, this paper introduces the IB loss [29] as the loss function of the sonar image recognition algorithm. When the majority of challenging samples belong to a more abundant category, adjusting the weights alone cannot improve the performance of the algorithm on other classes. IB loss addresses this issue by reducing the weights of these challenging samples and fine-tuning the decision boundary, allowing for a smoother and more balanced outcome. The optimization process of IB loss can be roughly divided into two steps. First, the algorithm is trained using a regular loss function until it reaches the vicinity of the optimal parameters. At this point, the loss function tends to zero. Then, IB loss is used for further training to smooth the decision boundaries. This step can improve the accuracy of the algorithm on classes with fewer samples and mitigate the problem of overfitting decision boundaries. The original MobileViT uses a cross-entropy loss function, whose gradient can be further calculated as:

(4) $$IB\left(x;w\right)=\frac{\partial }{\partial_{wk1}}L\left(y,f\left(x,w\right)\right)=\left(f_{k}-y_{k}\right)h_{1}.$$

The total gradient change is obtained by summing all the output gradients of the algorithm:

(5) $$\begin{array}{cc} IB(x;w) & =\sum_{k}^{K}\sum_{l}^{L}\left(f_{k}-y_{k}\right)h_{1}\\ & =\|f\left(x,w\right)-y_{1}\left\|\cdot \right\|h_{1}\|. \end{array}$$

To achieve equilibrium for each sample, the inverse of the gradient change is multiplied by the original loss to obtain a new loss function:

(6) $$L_{IB}\left(y,f\left(x,w\right)\right)=\frac{L(y,f(x,w))}{\|f\left(x,w\right)-y_{1}\left\|\cdot \right\|h_{1}\|}.$$

Then, this article continues by proposing balancing this loss with the number of categories, a method that balances the role of each category in the algorithm by reducing the weight of categories with large sample sizes.

(7) $$L_{IB}\left(y,f\left(x,w\right)\right)=\frac{\sum_{\left(x,y\right)\in D_{m}}\frac{\lambda_{k}L\left(y,f\left(x,w\right)\right)}{∥f\left(x,w\right)-y_{1}∥\cdot ∥h_{1}∥}}{m},$$

where

(8) $$\lambda_{k}=\frac{\sum_{k=1}^{K}n_{k}}{n_{k}}.$$

In this paper, we first trained 100 cycles using the cross-entropy loss function and then 50 cycles using IB loss.

## 4. Experimental Environment and Dataset

### 4.1. Experimental Environment

Our proposed algorithm is implemented with PyTorch 1.11.0. The entire algorithm is trained on a computer running Ubuntu 20.04 with Python 3.9. The training parameter settings are shown in Table 2.

### 4.2. Experimental Dataset

Because the acquired sonar images usually contain only one category of targets, the sonar target recognition method in this paper is applied for scenarios that target a single category of targets in the image. The sonar dataset used for the experiments in this paper is a fusion of three datasets. The first source is the underwater sonar images acquired by Sound Metrics, which is self-selected and established in this paper as the underwater sonar dataset USD; the second belongs to the publicly available sonar dataset SCTD, proposed by Zhang et al. [30]; and the third is the sonar image recognition dataset proposed by Matias et al. [31]. These several datasets contain a wealth of sonar data and provide the basis for the experimental studies in this paper.

**Figure 10:**
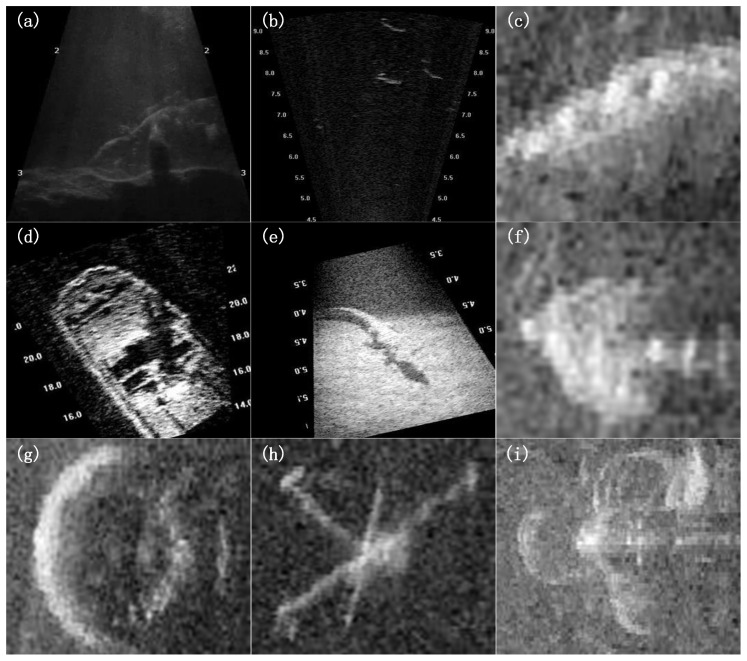
Some types of sonar images. (**a**) Person; (**b**) Fish; (**c**) Chain; (**d**) Shipwreck; (**e**) Alligator; (**f**) Drink carton; (**g**) Tire; (**h**) Valve; (**i**) Propeller.

Acquisition of underwater sonar data for real-world scenarios is greatly limited due to equipment constraints as well as national defense and military reasons. In this paper, the acquired sonar data are fused considering the real scenarios and classified into fourteen classes for the recognition algorithm in this paper, which are: persons, fish, shipwrecks, alligators, bottles, cans, chains, drink cartons, hooks, propellers, shampoo bottles, standing bottles, tires, and valves. Table 3 presents the dataset with its categories and their respective sizes in terms of total, training set, validation set, and test set. Some of the sonar data categories are displayed in Figure 10, where Figure 10a is a person, Figure 10b is fish, Figure 10c is a chain, Figure 10d is a shipwreck, Figure 10e is a alligator, Figure 10f is a drink carton, Figure 10g is a tire, Figure 10h is a valve, and Figure 10i is a propeller.

In this paper, data augmentation is realized by rotating, cropping, flipping, and other data enhancement operations on the original image, which to some extent avoids the overfitting problem that may occur during the training process.

## 5. Experimental Results and Analysis

**Figure 11:**
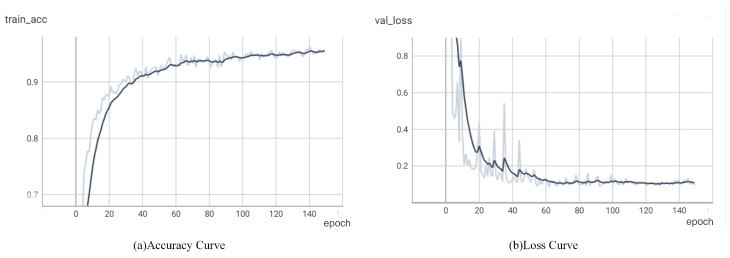
Accuracy and loss curves.

To train parameters that apply to underwater sonar images, this study conducted experiments to train the algorithm from scratch, and the weights were randomly initialized. In the experiment, the dataset was divided into a training set, testing set, and validation set with a ratio of 8:1:1. The input image size received by the algorithm was 224 × 224. Figure 11 shows the accuracy as well as loss curves of the model in this paper, where Figure 11a shows the accuracy curves and Figure 11b shows the loss curves, where the dashed line is the actual value and the solid line is the smoothed result of the actual value. As can be seen from the figure, the algorithm converges after about 80 epochs.

### 5.1. Ablation Experiment

To verify the effectiveness of the three improvement points in this paper, ablation experiments were first conducted in this paper. The three improvement points validated in the experiments are adding skip connections in the MobileViT block, introducing the improved Res2Net in the MV2 module and introducing the IB loss. The results are shown in Table 4, where a checkmark indicates improvement and a dash indicates no improvement with the original network structure retained.

The accuracy metric is the proportion of samples for which the class with the highest predicted probability by the model is consistent with the true label. It is calculated by comparing the predicted class indices with the true label indices and counting the proportion of correctly predicted samples to the total number of samples.

From Table 4, it can be seen that the baseline has a sonar image recognition accuracy of 92.30%. By simply adding two skip connections within the MobileViT block, the sonar recognition algorithm achieves an accuracy increase of 0.85%. Similarly, by incorporating the modified Res2Net network into MV2, the recognition accuracy improves by 1.08%. Furthermore, the accuracy of the algorithm increases by an additional 0.93% with the introduction of IB loss alone. These indicate that several improvement points of the algorithm in this paper are effective in enhancing the sonar image recognition accuracy of the model. Also, after adding two skip connections in the MobileViT block and a modified Res2Net network in MV2, the algorithm accuracy increases by 2.35%. Also, the algorithm accuracy increases by 2.33% after adding skip connections and introducing IB loss in the MobileViT block. Also, the addition of Res2Net to MV2 and the introduction of IB loss result in a 2.15% increase in algorithm accuracy. The last row is the algorithm of this paper. Finally, the recognition accuracy of the algorithm of this paper is 95.23%. The experimental results show that the various improvements implemented in this paper have contributed to the enhanced accuracy of the sonar image recognition algorithm to varying degrees.

### 5.2. Comparison Experiment

To further validate the performance of the proposed algorithm, this paper compares the performance of the proposed algorithm with other existing image recognition algorithms, including MobileViT, ResNet50 [32], MobileNetV3 [33], DenseNet121 [34], ViT [9], Swin_Transformer [11], as well as the algorithm in this paper. The comparison results are shown in Table 5. Five specific species are selected to be shown in the table, with several categories being persons, fish, shipwrecks, bottles, and alligators. The average accuracy represents the average detection result of the algorithm for the 14 categories of targets. The training strategy, as well as the epoch, are consistent across the algorithms, and all are trained until the algorithms converge.

As can be seen from Table 5, the algorithm in this paper performs optimally in terms of the average recognition accuracy of sonar images, which is 3.48% and 0.13% higher than the relatively better-performing algorithms ResNet50 and Swin_Transformer, respectively. The ViT algorithm applied to sonar images performs poorly and is not as effective as a general convolutional neural network. The average accuracy of the algorithm in this paper is improved by 2.93% over the baseline. The above analysis shows that the improved algorithm of this paper has a greater capability of capturing sonar features than other algorithms. From the five specific target categories shown in the table, it can be seen that for most of the targets, the algorithm in this paper obtains the highest recognition accuracy. Due to the relatively small target sizes of fish and alligators, the individual algorithms have lower recognition accuracies for both compared with the other categories. Although the recognition accuracy of the algorithm of this paper is slightly lower than the Swin_Transformer in the recognition of fish and alligators, and the effect needs to be improved, the gap is not too obvious. Additionally, it can be observed from Table 5 that the proposed algorithm performs the best in the category of persons and relatively average in the category of bottles. In conclusion, the proposed algorithm shows high performance in sonar image target recognition.

In addition, Table 6 presents a comparison of the parameter size and computational complexity of the proposed algorithm and other algorithms. Among them, flops represent the number of floating-point operations required during the model execution process. It is typically employed to indirectly gauge the temporal resource expenditure of a model. A higher flops value suggests that the model may necessitate a longer duration for data processing. As indicated by the data in Table 6, the baseline model manifests the lowest figures in both parameter quantity and flops, thereby highlighting its suitability under conditions of restricted computational resources. However, the model proposed in this study maintains a relatively low flops count while possessing a comparatively larger parameter volume, potentially enhancing its representational capability and overall model performance. Compared with models like ResNet50, ViT, and Swin_Transformer, the model introduced herein demonstrates considerable advantages in both the dimensions of parameter volume and flops. This indicates that the proposed model effectively reduces computational complexity without compromising on model capacity, subsequently showcasing significant superiority in inference speed and time cost. These characteristics imply that the proposed model is particularly apt for scenarios necessitating high-speed processing or where computational resources are limited.

## 6. Discussion and Limitations

In this study, we propose an optimized MobileViT algorithm for underwater sonar image target recognition and introduce several innovations and improvements. By enhancing the MobileViT algorithm, we successfully achieve the recognition accuracy of 95.23% for sonar image recognition tasks, indicating that our algorithm can more accurately identify and classify underwater targets. Furthermore, the algorithm demonstrates a precision of 0.9620 and a recall of 0.9594, along with a loss value of 0.199. It attains an F1 score of 0.9573 and achieves an AUC of 0.9576. Compared with the original ViT algorithm, our proposed MobileViT algorithm significantly reduces the computational resource requirements while maintaining high accuracy, making it more suitable for deployment on embedded systems or mobile devices with limited computational capabilities. We optimize the feature extraction mechanism by incorporating skip connections and improving the MV2 module, enabling the algorithm to capture key features more effectively, particularly when dealing with low-resolution and high-noise sonar images. Additionally, to address the issue of class imbalance in the training data, we propose a novel approach using the IB loss function. This strategy effectively tackles the challenge of class imbalance in the training data, ensuring good recognition performance for all target categories.

Although our proposed MobileViT algorithm has shown significant advantages in underwater sonar image target recognition tasks, it also faces some limitations and challenges. Firstly, while the algorithm performs well in sonar image recognition, its performance heavily relies on the quality and diversity of the training data. Insufficiently broad or biased training datasets may affect the algorithm’s generalization ability. Secondly, although our algorithm is more efficient compared with other high-accuracy algorithms, it still requires certain computational resources relative to extremely lightweight models. This may limit its real-time performance on low-end hardware. Furthermore, the introduction of skip connections in the MobileViT block and the incorporation of improved multi-scale convolutions in the MV2 module enhance the model’s performance. However, these enhancements also impose certain limitations on the algorithm’s transferability. This limitation arises from the increased complexity of advanced features in the algorithm, which consequently raises the difficulty of fine-tuning.

## 7. Conclusions

In this paper, an improved algorithm for underwater sonar image target recognition based on MobileViT was proposed. Considering that deep feature extraction layers may lose some important image features, this paper redesigned the skip connections to enhance the ability of the algorithm to capture crucial information from sonar images. Multiple scale features can effectively enhance the learning ability of the algorithm. Therefore, this paper introduced and redesigned the multi-scale convolutional Res2Net in the MV2 module, replacing the original single 1 × 1 convolution. This improvement increased the receptive field of the algorithm for sonar features. To address the issue of class imbalance in the sonar dataset, this paper introduced the IB loss function, which assigns different weights to samples based on their impact on training. The improved algorithm exhibited higher efficiency in utilizing effective features, resulting in improved accuracy in sonar image recognition. The experimental findings indicate that the proposed model improved in this study achieved the highest average accuracy of 95.23% among all models compared. This outcome not only confirms the efficacy of our improvement strategy but also attests to the feasibility and considerable potential for the application of the model in real-world scenarios. Furthermore, the lightweight nature of the algorithm has been maintained, allowing for easy deployment on embedded devices. We will be dedicated to expanding the diversity of sonar image datasets to enhance the generalization capabilities and robustness of the model. Through our research, the MobileViT algorithm has been improved. Additionally, the improved lightweight algorithm is suitable for embedded devices, enhances the ability to recognize minority class targets, and improves the processing level of sonar data. Our study has application value in fields such as underwater exploration, marine monitoring and security, underwater robots, and sonar monitoring systems.

## Figures and Tables

**Table 1:** Model test results.

Models of Different Sizes	Parameter Quantity (M)	Flops (G)
MobileViT-S	4.94	1.46
MobileViT-XS	1.93	0.74
MobileViT-XXS	0.95	0.27

**Table 2:** Parameter settings.

Parameters	Setting
Batch size	8
Learning rate	0.0002
Optimizer	Adam
Epoch	150

**Table 3:** Sonar dataset sizes.

Category	Total Size	Training Set Size	Validation Set Size	Test Set Size
Person	404	324	40	40
Fish	382	306	38	38
Shipwreck	831	665	83	83
Alligator	640	512	64	64
Bottle	449	361	44	44
Can	367	295	36	36
Chain	226	182	22	22
Drink cartons	349	281	34	34
Hook	133	107	13	13
Propeller	137	111	13	13
Shampoo bottle	99	81	9	9
Standing bottles	65	53	6	6
Tire	331	265	33	33
Valve	208	168	20	20
Total	4621	3711	455	455

**Table 4:** Ablation experiments of the improved MobileViT network.

Hopper Connection	Res2Net	IB Loss	Average Accuracy
−	−	−	92.30%
✓	−	−	93.15%
−	✓	−	93.38%
−	−	✓	93.23%
✓	✓	−	94.65%
✓	−	✓	94.63%
−	✓	✓	94.45%
✓	✓	✓	95.23%

**Table 5:** Comparison of accuracy in sonar image recognition for different algorithms. Bold indicates the best indicator in the same column.

Algorithm	Person	Fish	Shipwreck	Bottle	Alligator	Average Accuracy
Baseline	95.36%	92.21%	94.70%	89.53%	88.68%	92.30%
ResNet50	95.20%	90.01%	90.34%	85.78%	88.73%	91.75%
MobileNetV3	94.10%	84.30%	89.50%	86.30%	77.60%	89.06%
DenseNet121	92.00%	85.80%	93.90%	83.60%	83.53%	88.76%
ViT	86.77%	83.22%	82.59%	80.11%	83.78%	84.50%
Swin_Transformer	95.80%	**94.00**%	93.40%	90.50%	**94.60**%	95.10%
Ours	**96.80**%	93.33%	**95.10**%	**91.32**%	93.55%	**95.23**%

**Table 6:** Comparison of parameter quantity and efficiency.

Model	Parameter Quantity (M)	Flops (G)
Baseline	1.93	0.74
ResNet50	25.06	3.03
ViT	85.50	16.50
Swin_Transformer	28.50	4.00
Ours	2.90	1.20

## Data Availability

The availability of these data is limited, and they are used according to the license of the current study, so they are not publicly available.

## References

[1] He K., Zhang X., Ren S., Sun J. Deep residual learning for image recognition. Proceedings of the IEEE Conference on Computer Vision and Pattern Recognition.

[2] Xie S., Girshick R., Dollár P., Tu Z., He K. Aggregated residual transformations for deep neural networks. Proceedings of the IEEE Conference on Computer Vision and Pattern Recognition.

[3] Dong S., Wang P., Abbas K. (2021). A survey on deep learning and its applications. Comput. Sci. Rev..

[4] Janiesch C., Zschech P., Heinrich K. (2021). Machine learning and deep learning. Electron. Mark..

[5] Wang Z., Zhang S., Huang W., Guo J., Zeng L. (2021). Sonar image target detection based on adaptive global feature enhancement network. IEEE Sens. J..

[6] Shi P., Sun H., Xin Y., He Q., Wang X. (2023). SDNet: Image-based sonar detection network for multi-scale objects. IET Image Process..

[7] Gerg I.D., Monga V. (2021). Structural prior driven regularized deep learning for sonar image classification. IEEE Trans. Geosci. Remote Sens..

[8] Krizhevsky A., Sutskever I., Hinton G.E. (2017). ImageNet classification with deep convolutional neural networks. Commun. ACM.

[9] Dosovitskiy A., Beyer L., Kolesnikov A., Weissenborn D., Zhai X., Unterthiner T., Dehghani M., Minderer M., Heigold G., Gelly S. (2020). An image is worth 16x16 words: Transformers for image recognition at scale. arXiv.

[10] Meng L., Li H., Chen B.C., Lan S., Wu Z., Jiang Y.G., Lim S.N. Adavit: Adaptive vision transformers for efficient image recognition. Proceedings of the IEEE/CVF Conference on Computer Vision and Pattern Recognition.

[11] Liu Z., Lin Y., Cao Y., Hu H., Wei Y., Zhang Z., Lin S., Guo B. Swin transformer: Hierarchical vision transformer using shifted windows. Proceedings of the IEEE/CVF International Conference on Computer Vision.

[12] Liu Z., Hu H., Lin Y., Yao Z., Xie Z., Wei Y., Ning J., Cao Y., Zhang Z., Dong L. Swin transformer v2: Scaling up capacity and resolution. Proceedings of the IEEE/CVF Conference on Computer Vision and Pattern Recognition.

[13] Mehta S., Rastegari M. (2021). Mobilevit: Light-weight, general-purpose, and mobile-friendly vision transformer. arXiv.

[14] Dai Y., Zheng T., Xue C., Zhou L. (2023). MViT-PCD: A lightweight ViT-based network for Martian surface topographic change detection. IEEE Geosci. Remote Sens. Lett..

[15] Chen Z., Xie G., Deng X., Peng J., Qiu H. (2024). DA-YOLOv7: A Deep Learning-Driven High-Performance Underwater Sonar Image Target Recognition Model. J. Mar. Sci. Eng..

[16] Ruan F., Dang L., Ge Q., Zhang Q., Qiao B., Zuo X. (2022). Dual-Path Residual “Shrinkage” Network for Side-Scan Sonar Image Classification. Comput. Intell. Neurosci..

[17] Cheng Z., Huo G., Li H. (2022). A multi-domain collaborative transfer learning method with multi-scale repeated attention mechanism for underwater side-scan sonar image classification. Remote Sens..

[18] Liu X., Zhu H., Song W., Wang J., Yan L., Wang K. (2024). Research on improved VGG-16 model based on transfer learning for acoustic image recognition of underwater search and rescue targets. IEEE J. Sel. Top. Appl. Earth Obs. Remote Sens..

[19] Song Y., He B., Liu P. (2019). Real-time object detection for AUVs using self-cascaded convolutional neural networks. IEEE J. Ocean. Eng..

[20] Li X., Yang X., Ma Z., Xue J.H. (2023). Deep metric learning for few-shot image classification: A review of recent developments. Pattern Recognit..

[21] Li C., Li X., Chen M., Sun X. (2023). Deep learning and image recognition. Proceedings of the 2023 IEEE 6th International Conference on Electronic Information and Communication Technology (ICEICT).

[22] Gulzar Y. (2023). Fruit image classification model based on MobileNetV2 with deep transfer learning technique. Sustainability.

[23] Zhu Z., Lin K., Jain A.K., Zhou J. (2023). Transfer learning in deep reinforcement learning: A survey. IEEE Trans. Pattern Anal. Mach. Intell..

[24] Wang J., Li H., Huo G., Li C., Wei Y. (2023). Multi-modal multi-stage underwater side-scan sonar target recognition based on synthetic images. Remote Sens..

[25] Xu H., Bai Z., Zhang X., Ding Q. (2023). MFSANet: Zero-shot side-scan sonar image recognition based on style transfer. IEEE Geosci. Remote Sens. Lett..

[26] Long H., Shen L., Wang Z., Chen J. (2023). Underwater forward-looking sonar images target detection via speckle reduction and scene prior. IEEE Trans. Geosci. Remote Sens..

[27] Gao S.H., Cheng M.M., Zhao K., Zhang X.Y., Yang M.H., Torr P. (2019). Res2net: A new multi-scale backbone architecture. IEEE Trans. Pattern Anal. Mach. Intell..

[28] Hu J., Shen L., Sun G. Squeeze-and-excitation networks. Proceedings of the IEEE Conference on Computer Vision and Pattern Recognition.

[29] Park S., Lim J., Jeon Y., Choi J.Y. Influence-balanced loss for imbalanced visual classification. Proceedings of the IEEE/CVF International Conference on Computer Vision.

[30] Zhang P., Tang J., Zhong H., Ning M., Liu D., Wu K. (2021). Self-trained target detection of radar and sonar images using automatic deep learning. IEEE Trans. Geosci. Remote Sens..

[31] Valdenegro-Toro M., Preciado-Grijalva A., Wehbe B. (2021). Pre-trained models for sonar images. Proceedings of the OCEANS 2021: San Diego—Porto.

[32] He K., Zhang X., Ren S., Sun J. (2016). Identity mappings in deep residual networks. Proceedings of the Computer Vision–ECCV 2016: 14th European Conference.

[33] Howard A., Sandler M., Chu G., Chen L.C., Chen B., Tan M., Wang W., Zhu Y., Pang R., Vasudevan V. Searching for mobilenetv3. Proceedings of the IEEE/CVF International Conference on Computer Vision.

[34] Huang G., Liu Z., Pleiss G., Van Der Maaten L., Weinberger K.Q. (2019). Convolutional networks with dense connectivity. IEEE Trans. Pattern Anal. Mach. Intell..

